# MYST regulates DNA repair and forms a NuA4-like complex in the malaria parasite *Plasmodium falciparum*

**DOI:** 10.1128/msphere.00140-24

**Published:** 2024-04-02

**Authors:** Mohammad Kalamuddin, Ahmad Rushdi Shakri, Chengqi Wang, Hui Min, Xiaolian Li, Liwang Cui, Jun Miao

**Affiliations:** 1 Department of Internal Medicine, University of South Florida, Morsani College of Medicine, Tampa, Florida, USA; 2 Center for Global Health and Infectious Diseases Research, College of Public Health, University of South Florida, Tampa, Florida, USA; Indiana University School of Medicine, Indianapolis, Indiana, USA

**Keywords:** malaria, epigenetics, histone acetylation, DNA repair, chromatin remodeling

## Abstract

Histone lysine acetyltransferase MYST-associated NuA4 complex is conserved from yeast to humans and plays key roles in cell cycle regulation, gene transcription, and DNA replication/repair. Here, we identified a *Plasmodium falciparum* MYST-associated complex, PfNuA4, which contains 11 of the 13 conserved NuA4 subunits. Reciprocal pulldowns using PfEAF2, a shared component between the NuA4 and SWR1 complexes, not only confirmed the PfNuA4 complex but also identified the PfSWR1 complex, a histone remodeling complex, although their identities are low compared to the homologs in yeast or humans. Notably, both H2A.Z/H2B.Z were associated with the PfSWR1 complex, indicating that this complex is involved in the deposition of H2A.Z/H2B.Z, the variant histone pair that is enriched in the activated promoters. Overexpression of PfMYST resulted in earlier expression of genes involved in cell cycle regulation, DNA replication, and merozoite invasion, and upregulation of the genes related to antigenic variation and DNA repair. Consistently, PfMYST overexpression led to high basal phosphorylated PfH2A (γ-PfH2A), the mark of DNA double-strand breaks, and conferred protection against genotoxic agent methyl methanesulfonate (MMS), X-rays, and artemisinin, the first-line antimalarial drug. In contrast, the knockdown of PfMYST caused a delayed parasite recovery upon MMS treatment. MMS induced the gradual disappearance of PfMYST in the cytoplasm and concomitant accumulation of PfMYST in the nucleus, suggesting cytoplasm-nucleus shuttling of PfMYST. Meanwhile, PfMYST colocalized with the γ-PfH2A, indicating PfMYST was recruited to the DNA damage sites. Collectively, PfMYST plays critical roles in cell cycle regulation, gene transcription, and DNA replication/DNA repair in this low-branching parasitic protist.

**IMPORTANCE:**

Understanding gene regulation and DNA repair in malaria parasites is critical for identifying targets for antimalarials. This study found PfNuA4, a PfMYST-associated, histone modifier complex, and PfSWR1, a chromatin remodeling complex in malaria parasite *Plasmodium falciparum*. These complexes are divergent due to the low identities compared to their homologs from yeast and humans. Furthermore, overexpression of PfMYST resulted in substantial transcriptomic changes, indicating that PfMYST is involved in regulating the cell cycle, antigenic variation, and DNA replication/repair. Consistently, PfMYST was found to protect against DNA damage caused by the genotoxic agent methyl methanesulfonate, X-rays, and artemisinin, the first-line antimalarial drug. Additionally, DNA damage led to the relocation of cytoplasmic PfMYST to the nucleus and colocalization of PfMYST with γ-PfH2A, the mark of DNA damage. In summary, this study demonstrated that the PfMYST complex has critical functions in regulating cell cycle, antigenic variation, and DNA replication/DNA repair in *P. falciparum*.

## INTRODUCTION

It has been increasingly recognized that a multitude of developmental events in the malaria parasites is regulated by epigenetic mechanisms (1–8). The significance of epigenetics in malaria parasites is underlined by the relatively large number of genes predicted to be involved in chromatin structure, including enzymes adding the covalent histone post-translational modifications (PTMs)—the “writers,” proteins binding to these marks—the “readers,” and enzymes removing these marks—the “erasers.” *Plasmodium falciparum* histones are subject to a diverse array of PTMs, including acetylation, phosphorylation, and methylation, the abundance of which is dynamically regulated during the parasite’s life cycle (9–13). Acetylated histones are abundant euchromatic marks deposited by the histone acetyltransferases (HATs) PfGCN5 (14) and PfMYST (15).

In model organisms, HATs are present in large coactivator complexes to execute their diverse functions. For example, the NuA4 (Nucleosome Acetyltransferase of H4) complex contains the catalytic subunit Esa1 for the acetylation reaction (16–18). The yeast Esa1 and mammalian Tip60 are both members of the MYST family HATs. NuA4 is composed of 13 subunits, which are organized into the catalytic HAT module, the Tra1 subunit that binds to specific transcriptional factors, and an Eaf3/Eaf5/Eaf7 trimer with multiple functions (19–25). The mammalian Tip60 complex contains 17 proteins, representing the merge of two yeast complexes, the NuA4 complex and the chromatin remodeling complex SWR1/SRCAP (26–29). Thus, the Tip60 complex catalyzes both histone acetylation and the exchange of canonical H2A with the variant H2A.Z. The NuA4 and SWR1 complexes in yeast share four subunits (26–28, 30). The NuA4/Tip60 complexes regulate several pathways, including chromatin packaging, transcriptional activation, cell cycle, and DNA replication/repair (27). Consistently, PfMYST overexpression resulted in faster cell cycle progression (15). PfMYST and H4K8ac, a histone mark deposited by PfMYST, were enriched in the promoter of the activated *var* genes, suggesting that PfMYST also regulates antigenic variation (15, 31).

The NuA4/Tip60 complex has been implicated in DNA damage repair, a multi-step process. Tip60 activates ataxia-telangiectasia mutated kinase (ATM), a phosphatidylinositol 3-kinase-related kinase (PIKK), in response to DNA double-strand break (DSB). ATM rapidly phosphorylates H2A.X (at Ser139 in humans and Ser129 in yeast, called γ-H2A.X) and H2A (at Thr119 in humans and Ser122 in yeast, called γ-H2A) at the sites of DSB and these phosphorylations serve as the hallmark of DSB to recruit the NuA4 and SWR1 complexes (32–34). The subsequent H4/H2A acetylation by NuA4 and H2A.Z deposition by SWR1 promote chromatin relaxation, allowing the recruitment of DNA repair factors (26–29, 33, 35, 36). Notably, another important apicomplexan parasite, *Toxoplasma gondii*, contains two MYST homologs (A and B) and TgMYST-B could protect parasites from DNA damage partially by upregulating ATM and increasing γ-H2A.X (37). Multiple lines of evidence indicate that *P. falciparum* may use a similar mechanism to repair DNA damage. *P. falciparum* lacks the H2A.X variant, but the canonical *P. falciparum* H2A (PfH2A) is phosphorylated at Ser121 upon DNA damage to form γ-PfH2A foci in the nucleus (38). PfMYST acetylates H4 at K5, K8, K12, and K16 *in vitro*, and its overexpression increased resistance to DNA-damaging agents (15). Exposure to genotoxic agents and artemisinin led to increased levels of H4K8ac (39, 40). Interestingly, H4K8ac is enriched in intergenic regions of genes that may be involved in DNA damage response, leading to their upregulation (31, 41). Of note, artemisinin, also known to cause DNA damage in *P. falciparum* (42, 43), induced a similar transcriptional and epigenetic response to methyl methanesulphonate (MMS) (39). In Southeast Asia, artemisinin-resistant parasites have arisen with pre-disposing genetic factors, including mutations in DNA repair genes, which bestow the parasites with an enhanced ability to repair DNA damage and survive artemisinin treatment (43). These findings implicate PfMYST’s involvement in artemisinin resistance.

In this study, a NuA4-like and a SWR1-like complex with four common subunits were identified in *P. falciparum*. By transcriptomic analysis, we found that overexpressing PfMYST resulted in earlier expression of genes involved in DNA replication and cell cycle progression, as well as upregulation of genes related to antigenic variation and DNA repair. Interestingly, DNA damage led to the relocation of cytoplasmic PfMYST to the nucleus and colocalization of PfMYST with γ-PfH2A, the DSB mark. Finally, PfMYST overexpression caused a high level of basal γ-PfH2A and enhanced parasites’ tolerance to MMS, X-ray, and artemisinin treatment, and conditional knockdown of PfMYST led to a delayed growth recovery after MMS treatment, implying PfMYST’s participation in the repair of DNA damage resulting from various genotoxic agents.

## RESULTS

### PfMYST forms a NuA4-like complex in *P. falciparum*

*In silico* surveys in the *P. falciparum* genome identified PF3D7_1118600, PF3D7_1303800, PF3D7_0628600, PF3D7_0807000, and PF3D7_1422800 as homologs of ESA1, TRA1, EAF2/SWC4, YAF9, and ARP4 of the yeast NuA4 complex, respectively (15, 44, 45). Since we have experimentally identified an evolutionarily divergent PfGCN5-associated complex in *P. falciparum* (46), we wanted to employ a similar strategy to define the PfMYST complex(es). Using the PfMYST::PTP parasite line, in which the C-terminus of the endogenous *PfMYST* was tagged with a PTP tag in the 3D7 strain (15), we conducted tandem affinity purification (TAP) to isolate the PfMYST-associated complex. Since PfMYST is localized in both cytoplasm and nucleus (15), we performed the TAP procedure using nuclear extracts from 10^9^ asexual blood-stage parasites of either the PfMYST::PTP line or the wild-type (WT) 3D7 strain as the control. The purified protein complex was identified by liquid chromatography and tandem mass spectrometry (LC-MS/MS). The MS data were subjected to Significance Analysis of INTeractome (SAINT) using a threshold of probability above 95% and a false discovery rate (FDR) below 1% (47).

**Fig 1:**
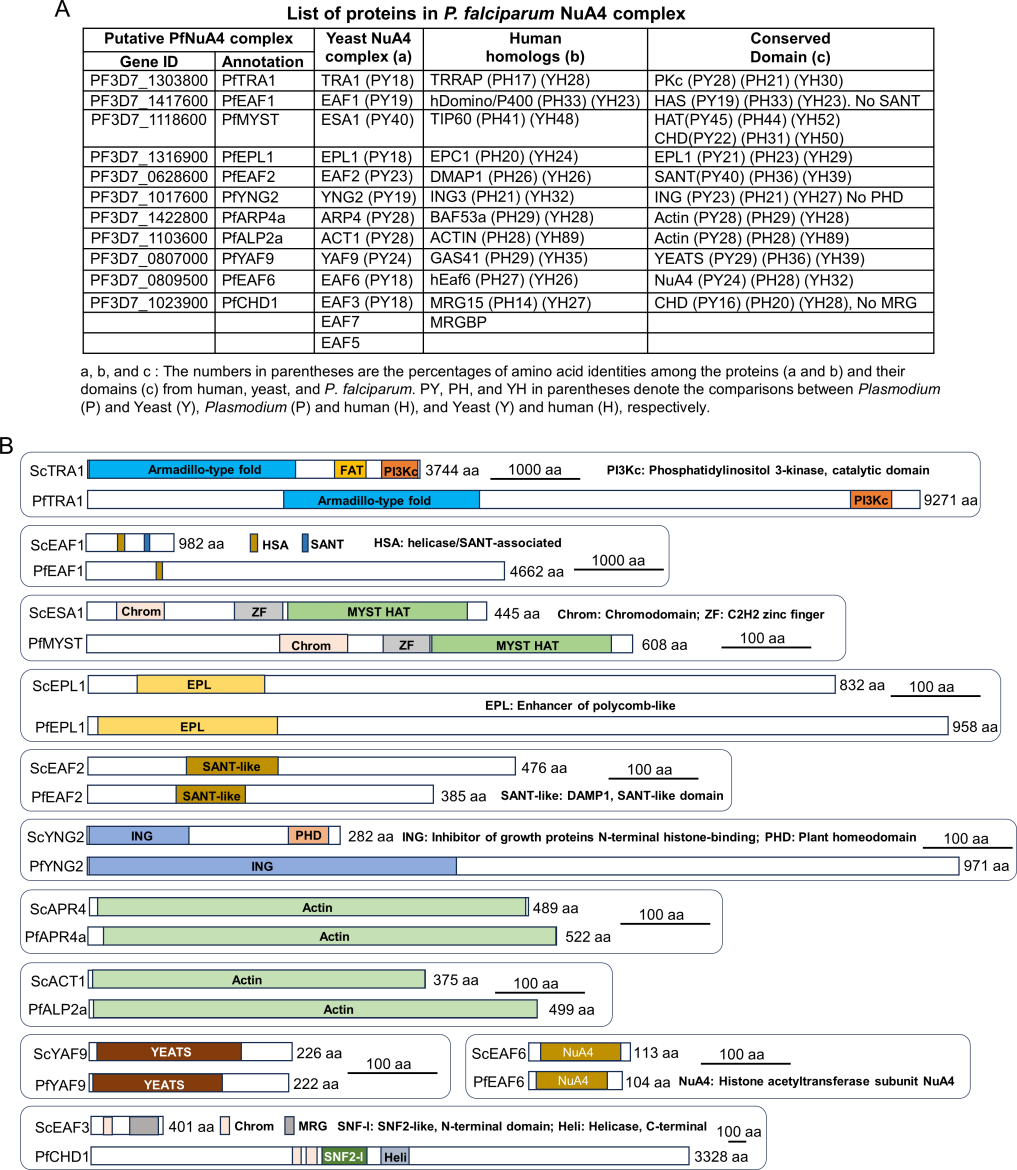
Identification of a NuA4-like complex in *P. falciparum*. (A) The identified proteins by TAP of PfMYST-associated proteins are listed along with their orthologs in yeast/human NuA4 complex. The identities among the orthologous proteins and their domains are shown in parentheses. PY, PH, and YH in parentheses denote the comparisons between *Plasmodium* (P) and yeast (Y), *Plasmodium* (P) and human (H), and yeast (Y) and human (H), respectively. (B) Schematic diagrams show the features (putative domains and protein size) of the identified subunits of PfNuA4 complex in *P. falciparum* along with their counterparts in yeast NuA4. The scale and domain name are included in each diagram.

Three biological replicates of TAP and LC-MS/MS consistently identified 21 PfMYST-associated proteins (Fig. 1A and B; Table S1). In addition to PfMYST, the *in silico* predicted TRA1, EAF2, YAF9, and APR4 homologs were among those identified proteins. By comparing the identifiable protein domains present in the identified proteins with the Saccharomyces cerevisiae NuA4 and human NuA4/Tip60 subunits, we tentatively assigned five identified proteins as homologs of the yeast NuA4 components: PfEAF1 (PF3D7_1417600), PfEPL1 (PF3D7_1316900), PfYNG2 (PF3D7_1017600), PfALP2a (PF3D7_1103600), and PfEAF6 (PF3D7_0809500) (Fig. 1A and B; Table S1). Additionally, PF3D7_1023900, annotated as a putative homolog of chromodomain-helicase-DNA-binding protein 1 (CHD1) (15, 44, 48), was detected in two replicates (Table S1). We assigned it the EAF3 homolog because of the shared chromodomain, although PfCHD1 is a much larger protein than the yeast EAF3 (Fig. 1A and B; Table S1). Two subunits (EAF5 and EAF7) of the yeast NuA4 complex could not be identified in PfMYST pulldowns. Altogether, PfMYST pulldowns identified 11 of the 13 components of the yeast NuA4 complex, indicating that *P. falciparum* harbors a NuA4-like complex, which we named the PfNuA4 complex (Fig. 1A). Other proteins identified from the PfMYST TAP include two subunits of the PfGCN5 complex (PfADA2 and PfPHD1), a MORC family protein (PF3D7_1468100), calmodulin (PF3D7_1434200), a putative protein mannosyltransferase (PF3D7_1010700), an ATP-dependent protease ClpY (PF3D7_0907400), an E3 ubiquitin ligase (PF3D7_0826100), karyopherin-α (PF3D7_0812400), and three membrane-bound proteins (RH4, RhopH2, and CLAG9), suggesting that the PfNuA4 complex may interact with various cellular targets (Table S1).

Despite relative conservation in the composition of the PfNuA4 complex, all subunits show low levels of sequence identity to their homologs in yeast and humans. In comparison, the identities between the yeast and human homologs in the NuA4/Tip60 complexes are generally higher than those between *P. falciparum* and yeast/humans (Fig. 1A; Table S1). Additionally, PfEAF1, PfYNG2, and PfCHD1 miss the SANT, PHD, and MRG domains compared to their respective yeast homologs (Fig. 1A and B). Furthermore, the predicted proteins PfTRA1, PfEAF1, and PfYNG2 are considerably larger than their yeast homologs (by 2.5, 4.7, and 3.4 times, respectively) (Fig. 1B). Therefore, the *P. falciparum* NuA4 complex diverged substantially from the NuA4 complex in model organisms.

### *P. falciparum* contains a conserved SWR1 complex

**Fig 2:**
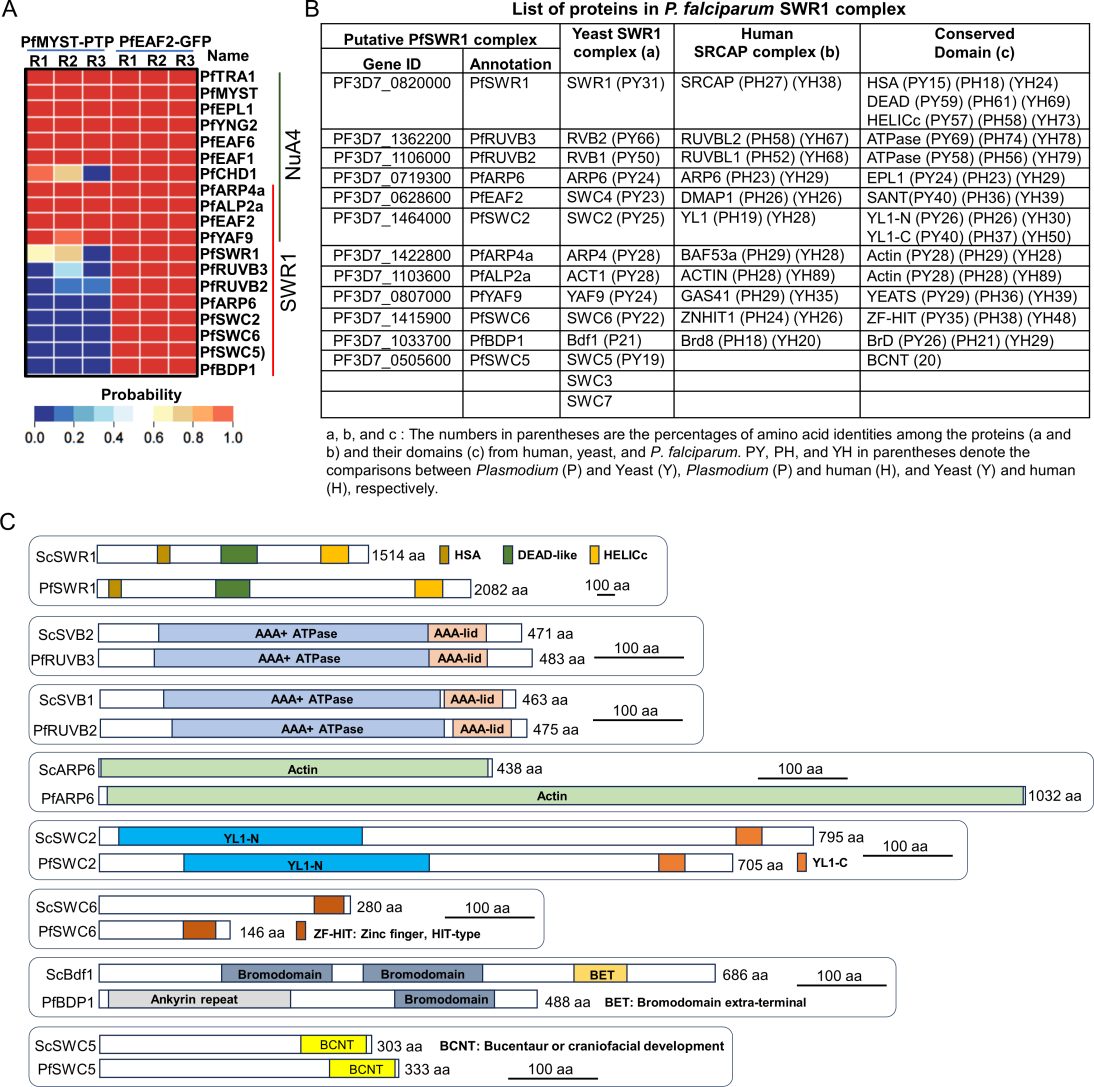
Validation of the PfNuA4 complex and identification of a SWR1-like complex. (A) Heatmap shows the identified proteins from the affinity purification of PfEAF2-associated proteins in PfEAF2::GFP parasites via GFP-trap beads compared to the NuA4-like subunits identified from TAP of PfMYST-associated proteins from PfMYST::PTP parasites. All 11 subunits of the PfNuA4 complex and 12 subunits of the PfSWR1 complex were identified by GFP-trap with three replicates (R1–R3). Four subunits are shared between the two complexes. The color range was shown based on the probability from SAINT analysis. (B) The identified PfSWR1 subunits are listed along with their homologs in the yeast/human SWR1/SRCAP complex. The identities among the orthologous proteins and their domains are shown in parentheses. PY, PH, and YH in parentheses denote the comparisons between *Plasmodium* (P) and yeast (Y), *Plasmodium* (P) and human (H), and yeast (Y) and human (H), respectively. (C) Schematic diagrams show the features (putative domains and protein size) of the identified subunits of PfSWR1 complex along with their homologs in yeast SWR1. The size scale and domain name are included in each diagram.

The yeast NuA4 and SWR1 complexes share four subunits, EAF2/SWC4, ARP4, ACT1, and YAF9 (26–28, 30). *In silico* prediction of the SWR1 complex in *P. falciparum* annotated five proteins PF3D7_0820000, PF3D7_1106000, PF3D7_1362200, PF3D7_1464000, and PF3D7_0719300 as homologs of SWR1, RVB1, RVB2, SWC2, and ARP6, respectively (44, 45). To confirm the PfNuA4 complex and identify the putative SWR1 complex in *P. falciparum*, we selected the shared subunit PfEAF2 between NuA4 and SWR1 as the target protein for protein pulldowns. By tagging PfEAF2 with the green fluorescent protein (GFP), we detected its nuclear localization during the IDC (Fig. S1). We used protein extracts from the PfEAF2::GFP parasites to perform protein pulldowns with the GFP-trap beads, followed by LC-MS/MS. Three replicates of protein pulldowns identified 34 proteins passing the same SAINT thresholds (Table S2), including all 11 subunits detected by the PfMYST TAP (Fig. 2A; Table S2). Similarly, the five predicted SWR1 subunits, PfSWR1, PfRVB1, PfRVB2, PfSWC2, and PFARP6, were also present in the PfEAF2 pulldowns (Fig. 2A and B; Table S2). Based on the domain structures, three genes PF3D7_0505600, PF3D7_1415900, and PF3D7_1033700 (PfBDP1) were annotated as the homologs of SWC5, SWC6, and Bdf1, respectively (Fig. 2B and C; Table S2). Thus, PfEAF2 pulldown identified 12 of the 14 SWR1 complex subunits, indicating that *P. falciparum* also contains an SWR1-like complex, which we name the PfSWR1 complex (26–28, 30) (Fig. 2; Table S2).

Like the PfNuA4 complex in *P. falciparum*, all subunits, and their domains in the PfSWR1 complex display much lower amino acid identities to their homologs in yeast or humans than the ones between yeast and humans (Fig. 2B). Compared to Bdf1, PfBDP1 only contains one bromodomain (Brd), lacking another Brd and BET (Fig. 2C). Furthermore, PfAPR6 is 2.4 times larger than its yeast homolog (Fig. 2C).

The yeast SWR1 complex is associated with H2A.Z and functions in the deposition of H2A.Z. Likewise, PfH2A.Z was also identified in the PfEAF2 pulldowns (Table S2), suggesting a similar function of the PfSWR1 complex. Strikingly, H2B.Z was also identified at a comparable abundance in the PfEAF2 pulldowns, implying that the PfSWR1 complex might deposit both histone variants. This contrasts with the detection of only H2B in the yeast SWR1 complex pulldowns (30, 49). Other proteins identified from the PfEAF2 pulldown include two other chromatin remodeling proteins (PF3D7_1104200 and PF3D7_0624600) annotated as SNF2L and ISWI, respectively (44, 48, 50), a high mobility group protein B3 (HMGB3), actin-like protein ALP2b, a protein containing poly D fragment (PF3D7_1441100), and a protein with unknown functions (PF3D7_0212100) containing a transcriptional activator LAG-3 family sequence (Table S2). This protein is localized in the nucleus and may function in chromatin regulation (48, 51).

### PfMYST regulates cell cycle, DNA replication/repair, and antigenic variation

**Fig 3:**
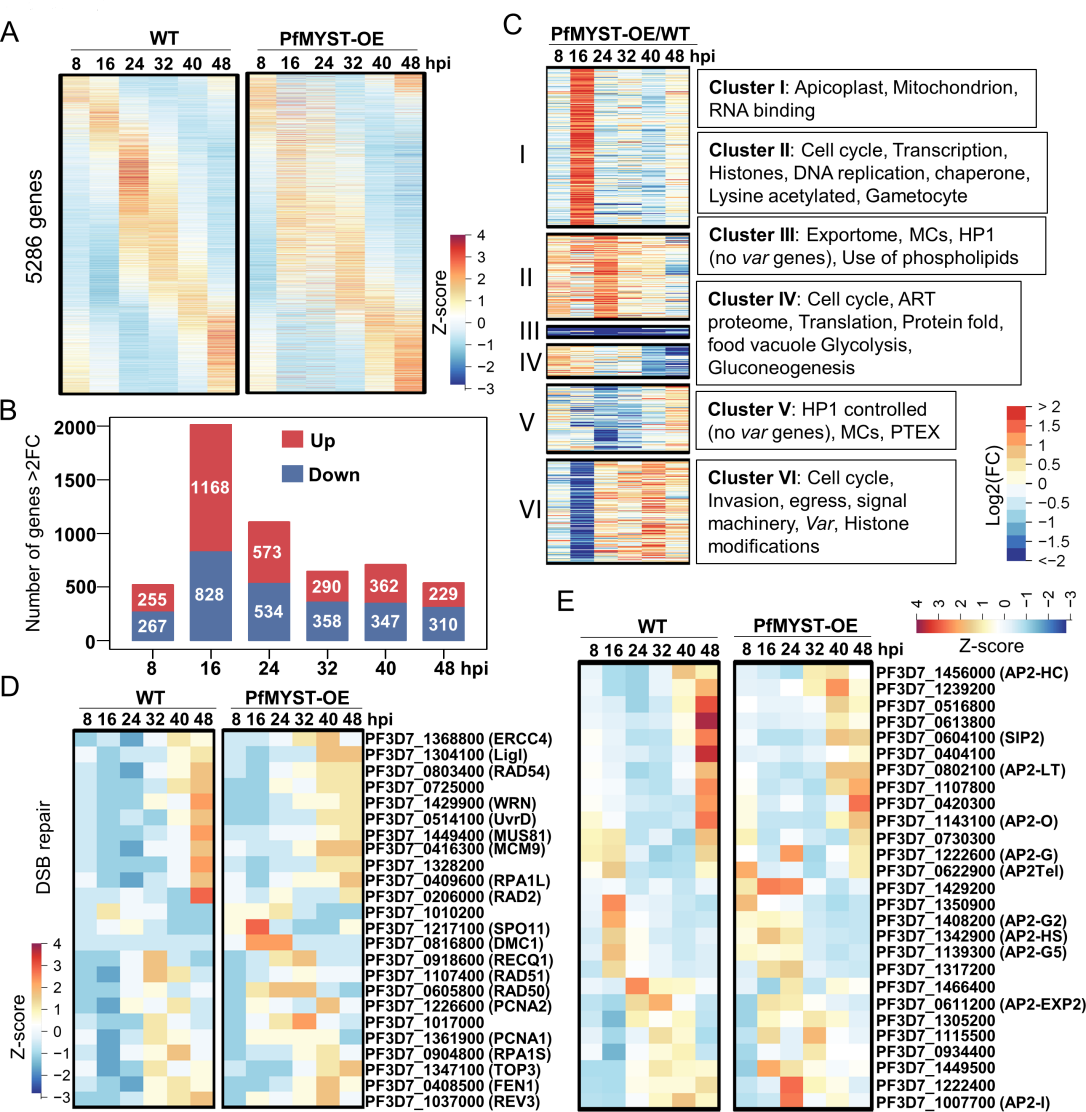
Global transcriptomic changes upon PfMYST overexpression. (A) The phaseograms of transcriptome from the WT 3D7, PfMYST-OE shows the disturbance of the cascade-like gene expression pattern in PfMYST-OE during IDC (from 8 to 48 hpi). (B) Number of genes with altered expression during IDC in PfMYST-OE compared to WT. The upregulated and downregulated genes are labeled in red and blue, respectively. (C) Heatmaps show the pattern (six clusters) of alteration in gene expression (fold change between PfMYST-OE and WT) upon PfMYST overexpression during IDC. The GO enrichment of each pattern is shown on the right side of the heatmaps. (D and E) Heatmaps show the expression of genes involved in DNA DSB repair (D) and AP2 domain-containing genes (E) in the WT (left) and PfMYST-OE (right) parasites during IDC (see details in Table S3).

Since PfMYST is an essential gene and refractory to gene disruption, we generated a PfMYST overexpression parasite line (PfMYST-OE) by introducing an additional copy of the full-length PfMYST expression cassette into the parasite genome (15). The PfMYST-OE parasites showed higher levels of acetylation of histone H4 at K5, K8, and K12, accompanied by substantially increased resistance of the parasites to DNA-damaging agents such as MMS (15). To explore global transcriptomic changes in the PfMYST-OE compared to the WT parasites, we selected six-time points during the IDC starting from a highly synchronized ring stage by short incubation (3 h) of mature schizont with red blood cells and performed microarray analysis using a custom-designed microarray (52). Compared with the WT parasites at each time point, PfMYST-OE parasites displayed significant changes in the phaseogram of gene expression (Fig. 3A; Table S3). By using >2-fold and adjusted *P* value < 0.05 as the threshold of significance, the most significant change occurred at the 16 h post-invasion (hpi), with 1,168 upregulated and 828 downregulated genes (Fig. 3B; Table S3). Based on the transcription patterns, K-mean clustering analysis divided the 3,210 genes with significantly altered expression into six clusters (Fig. 3C; Table S3). Cluster I included genes upregulated at 16 hpi. Gene ontology (GO) terms enrichment analysis showed specific enrichment of genes related to apicoplast and mitochondrion functions (Fig. 3C). Cluster II and Cluster VI genes were upregulated at the first five and the last four time points, respectively. Both clusters included genes related to cell cycle and histone modifications. Cluster II genes were also involved in transcription, DNA replication, and chaperone, whereas Cluster VI contains genes specific for invasion, egress, and signal machinery including PfPI3K (PF3D7_0515300). Interestingly, using the protein sequences of human ATM (hATM) and *T. gondii* ATM (TgATM) (37) to search their orthologs in *P. falciparum*, we identified PfPI3K at low identities to hATM (17.29%) and TgATM (14.35%), respectively, whereas the identity between TgATM and hATM is relatively higher (26.28%) (Table S3), suggesting PfPI3K might be the ATM in *P. falciparum*. A monoclonal antibody against hATM [2C1(1A1)] specifically recognized TgATM in the immunoblotting (37). To evaluate whether this antibody could also identify ATM in *P. falciparum*, we performed Western blots with extracted proteins from asexual parasites and found multiple reaction bands, indicating this antibody is not specific to PfATM (Fig. S2).

Genes upregulated in the first half of the IDC (Cluster IV) were involved in cell cycle, translation, protein fold, glycolysis, and hemoglobin digestion. In contrast, genes involved in export (exportome, Maurer’s clefts, and PTEX) and heterochromatin protein 1 (HP1) controlled genes (except the *var* genes) were downregulated during the entire IDC (Cluster III) or ring-trophozoite stages (Cluster IV). Earlier upregulation of genes involved in DNA replication and cell cycle in PfMYST-OE parasites is consistent with the more rapid growth phenotype (15). Notably, the total transcription of all *var* family genes showed a 34% increase in PfMYST-OE at 8 hpi, and almost all *var* genes at high transcription were upregulated upon PfMYST overexpression (Table S3; Fig. S3), consistent with the finding that PfMYST was involved in the activation of *var* genes (15). Although genes related to DNA repair were not enriched in the clustering analysis, 16, 13, 15, and 11 genes from 24, 31, 37, and 18 genes related to DSB repair, base-excision repair, nucleotide excision repair, and DNA mismatch repair (39, 53) were upregulated in PfMYST-OE in at least one of the studied time points, respectively, supporting elevated tolerance to DNA damage in PfMYST-OE (15) (Fig. 3D; Table S3). Furthermore, the expressions of 27 AP2-domain-containing proteins were substantially disturbed in PfMYST-OE (Fig. 3E; Table S3). Taken together, transcriptomic analysis indicates that PfMYST is involved in regulating cell cycle, energy metabolism (mitochondrion and glycolysis), hemoglobin digestion, stress response (protein folding and apicoplast), antigenic variation, and DNA replication/DNA repair.

### PfMYST protects the parasite against DNA damage

**Fig 4:**
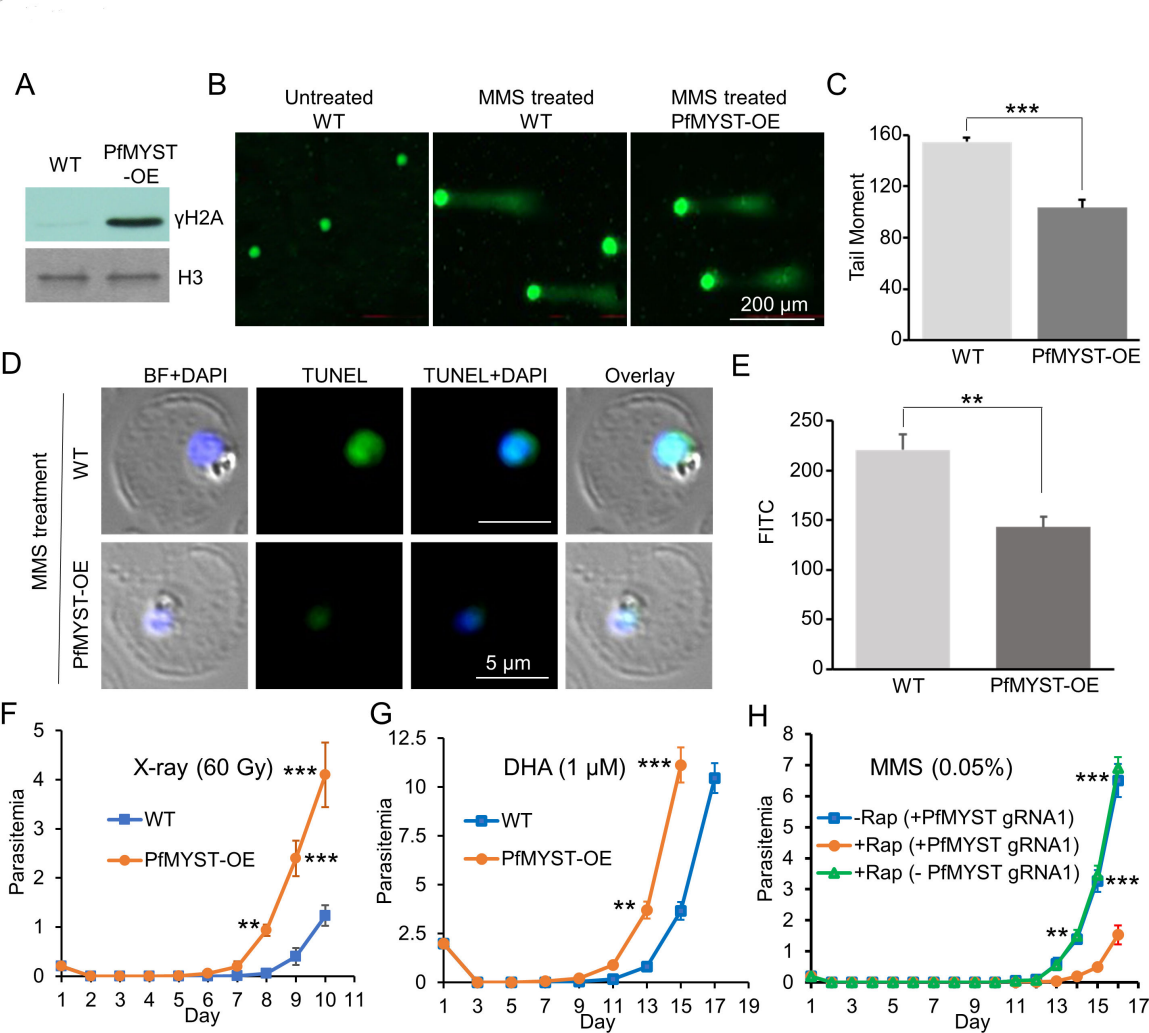
Overexpression of PfMYST protects parasites from DNA damage. (A) Western blots show a high level of γ-PfH2A in the PfMYST-overexpression parasites by mouse anti-γ-H2A.X monoclonal antibodies. (B) Representative pictures show the head and tail (fragmented DNA) of each parasite-infected RBC upon MMS treatment in the comet assays. (C) The average Tail Moment [tail length (µm) × % tail intensity/100] in PfMYST-OE was significantly lower than WT from three replicates of comet assays (***: *P* < 0.001, *t* test). (D) The representative pictures show the levels of fluorescein-dUTPs labeled DNA damage in the parasite-infected RBC upon MMS treatment in the TUNEL assays. DAPI was used for staining nuclear DNA. BF: bright field. (E) The overall Fluorescein (FITC) levels measured by flow cytometry were significantly lower than WT from three replicates of the TUNEL assay [**: *P* < 0.01(=0.0019), *t* test]. (F) The parasites recovered after the X-ray. PfMYST-OE reappeared earlier than WT. (G) The survival assays of PfMYST-OE after DHA treatment compared to WT. PfMYST-OE reappeared earlier than WT. (H) Knockdown of PfMYST by inducing dCas9-Sir2a expression via rapamycin (+Rap) and guiding dCas9-Sir2a to PfMYST promoter via PfMYST gRNA1 (+PfMYST gRNA1) led to a later parasite recovery (red line) compared to parasite lines without induction (−Rap, blue line) and with induction (+Rap) but without PfMYST gRNA1 (−PfMYST gRNA1, green line). Three biological replicates were performed for the recovery assays in (F–H). ** and *** denote *P* < 0.01 and *P* < 0.001, respectively.

A basal γH2AX in the active TgMYST-B-overexpression *T. gondii* but not in enzymatically dead TgMYST-B-overexpression parasites was found without any induction by DNA damage (37). In *P. falciparum*, DNA damage induced PfH2A phosphorylation on serine 121, the mark at the sites of DSB (38). Similarly, we discovered a high level of basal γH2A in PfMYST-OE parasites (Fig. 4A), suggesting that PfMYST is involved in the upregulation of H2A phosphorylation and partially explaining why PfMYST-OE parasites were more resistant to DNA damage (15). To further define the role of PfMYST in protection against DNA damage, we conducted comet and TUNEL assays to assess the level of DNA damage upon MMS treatment in the PfMYST-OE and WT parasites (Fig. 4B through E). Compared to WT parasites, MMS caused significantly less DNA damage in PfMYST-OE, as measured by the comet assay (Fig. 4B and C, *P* < 0.0001, *t* test). Similarly, the TUNEL assay showed significantly less DNA labeling in PfMYST-OE than in WT parasites (Fig. 4D and E, *P* = 0.0019, *t* test). We then tested whether PfMYST-OE could protect against DNA damage by X-ray irradiation. After ring-stage (0–6 hpi), parasites at 0.2% parasitemia were radiated with a dose of 60 Gy, PfMYST OE parasites first appeared in culture on day 6 and reached 1% parasitemia on day 8 (Fig. 4F). In contrast, WT parasites appeared on day 8 and reached 1% parasitemia on day 10 after the same X-ray irradiation (Fig. 4F). Since artemisinin can cause DNA damage via reactive oxygen species (42, 54), we tested whether PfMYST-OE could survive better than WT parasites upon artemisinin treatment. In the parasite recovery assay, early rings were treated with 1 µM of dihydroartemisinin (DHA) for 12 h. Live PfMYST-OE parasites were detected on day 9 and reached 1% on day 11 compared to 2-day delays in the WT parasite (Fig. 4G), consistent with a recent report showing that upregulation and downregulation of PfMYST by conditional CRISPR activation (a) and interference (i) systems led to earlier and later recovery of parasite growth, respectively (55). Likewise, the knockdown of PfMYST by CRISPRi also caused the later recovery of parasite growth upon MMS treatment (Fig. 4H; Fig. S4).

### DNA damage led to the relocation of PfMYST from the cytoplasm to the nucleus

**Fig 5:**
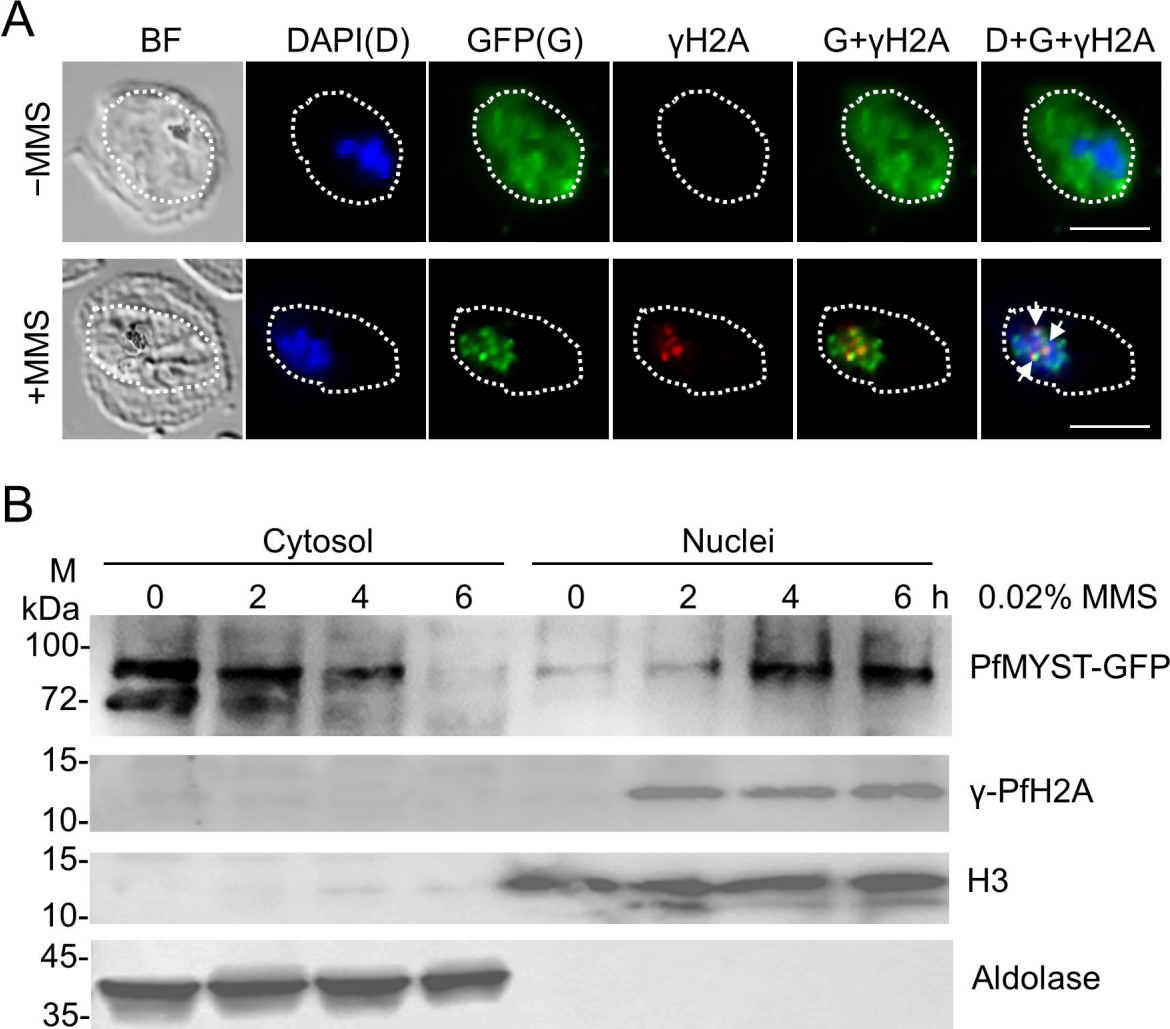
PfMYST was translocated from cytosol to the nucleus upon DNA damage. (A) The representative pictures show the GFP and γ-PfH2A distribution in the PfMYST::GFP parasites with (+) and without (−) MMS treatment (0.02%, 6 h). PfMYST-GFP was accumulated in the parasite nucleus after MMS treatment and was partially colocalized with γ-PfH2A (white arrows). DAPI was used for staining nuclear DNA. The parasite boundary was labeled by dashed lines. BF: bright field. The size scale bar is 5 µm. (B) Western blots show the time-dependent translocation of PfMYST from the cytosol to the nucleus upon DNA damage. Before MMS treatment (0 h), most PfMYST was localized in parasite cytosol and existed as two versions (short and long), whereas only the long version of PfMYST was found in the parasite nucleus. Upon MMS treatment, both versions of PfMYST gradually disappeared in the cytosol; meanwhile, the long version of PfMYST accumulated in the nucleus. γ-PfH2A appeared 2 h after MMS treatment. Aldolase and H3 were used as marks of cytosolic and nuclear fractions. Three biological replicates were performed for the translocation of PfMYST in (B).

To further demonstrate PfMYST’s involvement in DNA repair, we treated the PfMYST::GFP, a parasite line in which endogenous PfMYST was tagged by GFP (15), at the trophozoite stage with 0.02% MMS for 6 h and the GFP signals became more concentrated in the nuclei compared to the GFP signals in both cytosol and nucleus of the untreated parasites (Fig. 5A). γ-PfH2A puncta appeared in the nuclei of treated parasites and partially colocalized with PfMYST (Fig. 5A). Next, Western blots were performed to confirm these observations. MMS-treated parasites were fractionated into the cytosolic and nuclear compartments, as confirmed by immunoblotting of the extracts with antibodies against the nuclear marker histone H3 and the cytoplasmic marker aldolase (Fig. 5B). In agreement with the previous report (15), PfMYST expression uses two different start codons, resulting in a long and a short version (Fig. 5B). Before MMS treatment, the majority of PfMYST was in the cytosol. Interestingly, the short version of PfMYST was only detected in the cytoplasm of the parasite. Shortly after MMS treatment, both versions of PfMYST gradually decreased in the cytoplasm, with a concomitant increase of the long PfMYST version in the nucleus (Fig. 5B). Meanwhile, γ-PfH2A appeared 2 h after MMS treatment. Given that stress conditions in *P. falciparum* led to a global translational shutdown, the increase of PfMYST in the nucleus is more likely the result of the cytoplasm-nucleus shuttling of PfMYST.

## DISCUSSION

In this study, we first reported the successful identification of a NuA4-like and a SWR1-like complex in *P. falciparum* and then investigated the function of the PfNuA4 complex’s catalytic subunit, PfMYST, in gene regulation by transcriptomic analysis and DNA repair by analyzing DNA damage responses in the PfMYST-overexpression or PfMYST-knockdown parasite lines.

Compared to the distinct PfGCN5 complex that shared only two subunits (GCN5 and ADA2) with the SAGA complex in model organisms (46), the PfNuA4 and PfSWR1 complexes are relatively conserved composition-wise, although the overall identities between the *P. falciparum* proteins and putative yeast/human homologs were low, and mostly are restricted to identifiable protein domains (Fig. 1 and 2). Cryo-electron microscopy structure studies of the yeast NuA4 complex have revealed two major modules: the transcription activator-binding TRA module (Tra1, Actin, Arp4, Eaf2, Eaf1, and Epl1 C-terminus) and catalytic HAT module (piccolo complex: Esa1, Epl1 N-terminus, Yng2 and Eaf6) (19–25). Eaf5/7/3 (TINTIN module) formed a subcomplex and connected the HAT and TRA modules. A large portion of Eaf5/7/3 is outside the NuA4 complex and plays a role in RNA polymerase II-coupled nucleosome recycling (56, 57). Thus, the weak association between Eaf5/7/3 and NuA4 major modules could be why we did not identify these subunits in the PfMYST pulldown. Another possibility is that *P. falciparum* does not encode these subunits because no EAF5/7 homologs were identified *in silico* surveys. Furthermore, TRA1 is a shared subunit of NuA4 and SAGA in model organisms from yeast and plants to humans, but in *P. falciparum*, it was only identified in the PfNuA4 complex (46).

We identified 12 of the 14 subunits of the yeast SWR1 complex in *P. falciparum,* including the four shared subunits between NuA4 and SWR1. Subunit conservation and association with PfH2A.Z suggest that the PfSWR1 complex might function similarly in H2A.Z deposition (26–28, 30). Similarly, 12 SWR1 subunits were also identified in *Trypanosoma brucei* (58). Surprisingly, besides PfH2A.Z, PfH2B.Z was also identified in the pulldown, suggesting that PfSWR1 might deposit PfH2A.Z/PfH2B.Z instead of H2A.Z/H2B in the model organisms. H2B.Z is unique in apicomplexan parasites (59). PfH2A.Z/PfH2B.Z double-variant nucleosomes located in the AT-rich intergenic regions (e.g., promoters) are associated with gene activation (60, 61). Similarly, TgH2A.Z/TgH2B.Z dimer localizes with activate mark H3K4me3 in promoter/transcriptional start site regions of active genes and the gene bodies of silent genes (62). Two recent reports showed that PfH2A.Z was colocalized with PfARP4a and its knockdown resulted in the depletion of PfH2A.Z and reduced H3K9ac at the upstream regions of genes (63) and five mutations of N-terminal lysines of TgH2B.Z to arginines led to defects of parasite growth and virulence, more sensitive to DNA damage, and increase of differentiation to bradyzoites (64). The SWR1 complex in these parasites might be specifically involved in the regulation of the abovementioned processes.

Transcriptomic analysis of PfMYST overexpression provided clues on PfMYST’s function in regulating cell cycle, *var* gene expression, and DNA replication/repair. Intriguingly, of the HP1-controlled heterochromatic genes, only *var* family genes were upregulated, whereas other multigene families (e.g., rifin, PHIST, and Pfmc-2TM) were downregulated upon PfMYST overexpression. This phenomenon agrees well with the interesting findings that PfH2A.Z/PfH2B.Z double-variant nucleosomes are only enriched in the promoter of the active *var* genes (61). Moreover, both PfMYST and H4K8ac are enriched in the promoter of the activated *var* genes (15, 31), suggesting that PfNuA4 and PfSWR1 are specifically involved in the regulation of antigenic variation in *P. falciparum*. Interestingly, a recent study showed that PfH2A.Z/PfH2B.Z were specifically enriched in the heterochromatin of female gametocytes (65). Whether PfNuA4 and PfSWR1 are involved in this regulation remains to be determined. We also noticed that many genes encoding apicoplast-localized proteins were upregulated at the ring stage. Since apicoplast is essential for parasite survival in response to stress conditions (66, 67), PfMYST may increase stress tolerance by enhancing apicoplast functions. Since the above transcriptomes were derived from the PfMYST-overexpression parasite line in which PfMYST was constitutively over-expressed in the parasites, the observed differential gene expression could be both direct and indirect or consequential responses to PfMYST overexpression.

A basal γ-H2A in the PfMYST-overexpression parasite line and assessment of the DNA damage by both comet and TUNEL assays confirmed that PfMYST is involved in DNA repair after MMS treatment. Additional evidence was derived from the survival assays which displayed that overexpression of PfMYST protected parasites against the DNA damage caused by X-ray and DHA treatment and knockdown of PfMYST led to later recovery of parasite growth upon MMS treatment. The disappearance of cytosolic PfMYST and increased level of nuclear PfMYST after DNA damage suggest cytoplasm-nuclear shuttling of PfMYST in *P. falciparum*. Given the partial colocalization of the DSB signals (γ-H2A) with PfMYST, it is reasonable to assume that PfMYST is recruited to the DSB sites. Similar cytosolic and nuclear localizations of MYST (MYST-A and MYST-B) were found in *T. gondii* (37), but TgMYST did not show noticeable relocation after MMS treatment. Furthermore, because the shorter PfMYST was only found in the cytoplasm, whether there is a functional division between the shorter and longer versions of PfMSYT remains to be determined.

## MATERIALS AND METHODS

### Parasite culture

The *P. falciparum* strain 3D7 and its genetically modified clones were cultured at 37°C in a gas mixture of 5% CO_2_, 3% O_2_, and 92% N_2_ with type O^+^ red blood cells (RBCs) at 5% hematocrit in RPMI 1640 medium supplemented with 0.5% Albumax II (68).

### Genetic manipulation of *PfEAF2*

A single-crossover recombination approach was used to tag PfEAF2 with GFP in the 3D7 parasite (46, 69). The C-terminus of PfEAF2 was PCR amplified with primers F1 and R1 (Table S4). The PCR fragment was cloned into the modified pBluescript SK to fuse with GFP and pDT 3′ UTR as described earlier (15, 70). This cassette was then subcloned into pHD22Y at the *Bam*HI and *Not*I sites to produce pHD22Y/PfEAF2-GFP (70) (Fig. S1). Parasite transfection was done using the RBC loading method (71). Single clones of parasites with stable integration of the construct were obtained by limiting dilution (72). Correct integrations of plasmids into the parasite genome were screened by integration-specific PCR with primer F2 and R2 (Table S4; Fig. S1).

### Purification of protein complexes

TAP was performed using the PTP-tagged PfMYST parasite line according to the published method (46, 73). Briefly, 10^9^ parasites were lysed in the hypotonic buffer. The resultant pellet (nucleus) was lysed in PA150 buffer (150 mM KCl, 20 mM Tris-HCl, pH 7.7, 3 mM MgCl_2_, 0.5 mM DTT, and 0.1% Tween 20) containing a protease inhibitor cocktail (Roche). The lysate was centrifuged, and the supernatant was incubated with IgG agarose beads (GE Healthcare) at 4°C for 2 h. The beads were then equilibrated with the TEV buffer (PA150 plus 0.5 mM EDTA) and incubated with 150 U of TEV protease overnight at 4°C. The supernatant was incubated with the anti-protein C beads for 2 h at 4°C and the bound proteins were eluted. For the single-step pulldown of GFP-tagged PfEaf2, GFP-trap (Cat# gta-20, RRID:AB_2631357, Chromotek) beads were used with lysates from 10^9^ parasites according to the manufacturer’s protocol.

### Mass spectrometry

The proteins in the elution were separated briefly in a 10% Bis-Tris SDS-PAGE gel for 10 min. Proteins in gel were excised, in-gel digested, and analyzed by nano-LC/MS/MS using a Waters NanoAcquity HPLC system interfaced to a Q Exactive Hybrid Quadrupole-Orbitrap Mass Spectrometer (Thermo Scientific) (74). Peptides were loaded on a trapping column and eluted over a 75-µm analytical column at 350 nL/min. MS and MS/MS were performed at 70,000 FWHM and 17,500 FWHM resolutions, respectively. The 15 most abundant ions were selected for MS/MS. Parasite proteins were identified by searching the Uniprot *P. falciparum* protein database (v01/2014). Data were filtered at 1% protein and 0.2% peptide FDR, and at least two unique peptides per protein. Mascot DAT files were parsed into the Scaffold software for validation and filtering to create a non-redundant list per sample.

### Immunoﬂuorescence assay

Immunoﬂuorescence assay (IFA) was performed as described (75, 76). The parasitized RBCs were ﬁxed with 4% paraformaldehyde and 0.0075% glutaraldehyde, followed by quenching with 50 mM glycine. Fixed cells were permeabilized with 0.5% Triton X-100 and blocked in 3% BSA and incubated with primary antibodies: mouse monoclonal anti-γ-H2A.X (1:200; clone JBW301, Sigma-Aldrich, AB_2924829, USA) or goat anti-GFP antibodies (1:200; ab6673; Abcam, RRID:AB_305643, USA) and secondary antibodies: Alexa Fluor 488-donkey anti-goat IgG antibodies (ab150129, RRID:AB_2687506, Abcam, USA) or Alexa fluor 647-rabbit anti-mouse antibodies (ab150127, Abcam, USA). Nuclei were stained with DAPI (Invitrogen). Images were captured using an epifluorescence microscope (Nikon Eclipse Ni, USA).

### Subcellular fractionation and Western blot

Subcellular fractionation was performed using an established method (69). Briefly, parasites were under a freeze-thaw process followed by centrifugation. The supernatant was harvested as the cytoplasmic fraction. The pellet was then suspended in RIPA Buffer followed by centrifugation, and the supernatant was used as the nuclear fraction. Goat anti-GFP antibodies (1:2,500, ab6673, Abcam, RRID:AB_305643, USA), mouse monoclonal anti-γ-H2A.X (1:2,500; clone JBW301, Sigma-Aldrich, AB_2924829, USA), and hATM [1:1,000, 2C1(1A1), ab78, Abcam, RRID:AB_306089], rabbit anti-histone H3 antibodies (1:1,000 dilution; Millipore), and rabbit anti-Plasmodium aldolase antibodies (1 µg/mL, ab207494, Abcam, USA) were used as primary antibodies. HRP-conjugated goat anti-rabbit IgG (1:5,000, Millipore) or rabbit anti-goat or mouse IgG (ab6741, RRID:AB_955424, ab6728, RRID:AB_955440, Abcam, USA) was used as the secondary antibodies. The results were visualized with the ECL detection system (Clarity Max, Bio-Rad).

### Comet assay

The extent of DNA damage in parasites was assessed by migration of fragmented DNA called comet assay (42, 43, 77). We performed a single-cell gel electrophoresis alkaline comet assay (Comet Assay Kit, R&D Systems, Catalog #4250-050-K) according to the manufacturer’s manual. Briefly, trophozoites were treated with 0.02% MMS (Sigma, USA) for 6 h. Then, parasites were mixed with 1% molten Low Melting Agarose. About ~50 µL mixture was added to wells on slides (CometSlide) for single-cell gel electrophoresis. The migrated DNA fragments were precipitated, dried, and then stained with SYBR green. Images were taken under a fluorescent microscope (Nikon Eclipse). The percentage (%) of DNA intensity in the tail compared to the total intensity of both head and tail and Tail Moment [tail length (µm) × % tail intensity/100] were calculated by ImageJ software.

### TUNEL assay

DNA damage was also assessed by Terminal deoxynucleotidyltransferase-mediated dUTP Nick End Labelling (TUNEL), in which a fluorophore-conjugated modified nucleotide is linked to 3′-OH end of the fragmented (damaged) DNA (38, 78). This assay was performed using the *In Situ* Cell Death Detection Kit, Fluorescein (Roche, Cat No. 11684795910, Version 18) per the manufacturer’s instructions. Briefly, early trophozoites were treated with 0.02% MMS for 6 h, and then fixed (2% paraformaldehyde), permeabilized (0.1% Triton X-100), and reacted with TUNEL reaction mixture. Smears were prepared and mounted with an antifade reagent containing DAPI (Invitrogen Molecular Probes). Images were captured using an epifluorescence microscope (Nikon Eclipse Ni, USA). Flow cytometry (Becton Dickinson LSR II) was used for analyzing the mean FITC (green) of DAPI-positive cells. 10,000 cells were sampled for each condition.

### X-ray irradiation of parasites

For DNA damage recovery assay, 0.2% early rings were treated with 60 Gy (6,000 rad) X-ray irradiation using an XRAD160 X-ray irradiator (Precision) at 160 kV, 19 mA, and 2 mm A1 filter as the established methods (38, 78, 79). Population recovery was measured by daily Giemsa staining.

### Growth recovery assays upon DHA or MMS treatment

The parasite growth recovery assay was performed according to the established methods (54, 55, 80–82). Early ring-stage (0–6 h) of PfMYST-overexpression parasites (15) at 2% parasitemia or Dicre-dCas9-Sir2a parasites with PfMYST gRNA1 (55) at 0.2% parasitemia were treated with 1 µM DHA for 12 h or 0.05% MMS for 6 h, respectively. Parasitemia was checked daily by Giemsa staining.

### RT-qPCR

The Quick-RNA MiniPrep Kit (Zymo Research) was used for extracting total RNAs from the CRISPRi parasite line expressing gRNA1 targeting PfMYST 5′ UTR (55). Reverse transcriptase and real-time PCR were performed by using SuperScript III RT (Invitrogen) and Faststart Universal SYBR green master mix (Roche). The relative expression of *PfMYST* gene in the Rap-induced or dimethyl sulfoxide (DMSO)-treated parasite line was normalized to seryl-tRNA synthetase (PF3D7_0717700) and calculated by the established method (83). Three biological replicates were performed.

### Transcriptome analysis

Expression microarrays were performed based on a custom-designed expression microarray designed by Roche NimbleGen (Madison, WI) (52). Highly Synchronized parasites were obtained by incubating mature schizonts with RBCs for 3 h in order to collect parasites from ring to schizont stages at six-time points with 8 h intervals (72). Total RNA from parasites was harvested by using the ZYMO RNA purification kit. RNA was amplified and labeled with Cy5 or Cy3 using an Amino Allyl MessageAmp II aRNA Amplification Kit (Ambion, Austin). The expression levels and the differential expression were calculated by the criteria of ≥2-fold change (FC). Additionally, maximum likelihood estimation was performed like the analysis in the previous study (67). Subsequently, the final *P* value was calculated based on the fitted Gaussian distribution of the microarray data, and the false discovery rate was employed to adjust the *P* values. We identified differential expressions using an absolute log2(FC) greater than one and a *P* adjustment lower than 0.05. The sine wave model was utilized to model the gene expression timing (84) and the phaseogram of the transcriptomes was plotted according to the established methods (46).

### Statistical analysis

For all experiments, three independent biological replicates were performed. The results are presented as mean ± SD. Results were considered significant if *P* < 0.05 as established by ANOVA and *t* test, and the respective analysis was shown in the figure legends.

## Data Availability

Microarray data were submitted to the NCBI GEO repository (accession number GSE245596). The available mass spectrometry proteomics data have been deposited to the ProteomeXchange Consortium via the PRIDE (85) partner repository with the data set identifier PXD046547.

## SUPPLEMENTAL MATERIAL

Supplemental figures msphere.00140-24-s0001.pdf Figures S1-S4.

Table S1 msphere.00140-24-s0002.xlsx List of identified proteins from TAP of PfMYST-associated proteins.

Table S2 msphere.00140-24-s0003.xlsx List of identified proteins from GFP-trap of PfEAF2 associated proteins.

Table S3 msphere.00140-24-s0004.xlsx Expression microarray of PfMYST-OE compared to WT during IDC.

Table S4 msphere.00140-24-s0005.xlsx Primers used in this study.

ASM does not own the copyrights to Supplemental Material that may be linked to, or accessed through, an article. The authors have granted ASM a non-exclusive, world-wide license to publish the Supplemental Material files. Please contact the corresponding author directly for reuse.
