# Supplementary material for: MYST regulates DNA repair and forms a NuA4-like complex in the malaria parasite Plasmodium falciparum
Source: mSphere. 2024 Apr 2;9(4):e00140-24. doi: 10.1128/msphere.00140-24 (PMC11036802; doi:10.1128/msphere.00140-24)
Supplement: Supplemental figures — Figures S1-S4. [file msphere.00140-24-s0001.pdf]

# Supplementary Materials

**MYST regulates DNA repair and forms a NuA4-like complex in the malaria parasite *Plasmodium falciparum***

Mohammad Kalamuddin[a#§], Ahmad Rushdi Shakri[a#], Chengqi Wang[b#], Hui Min[a], Xiaolian Li[a], Liwang Cui[a,b], Jun Miao[a,b]*

[a]Department of Internal Medicine, Morsani College of Medicine, University of South Florida, 3720 Spectrum Blvd, Tampa, FL 33612, USA.

[b]Center for Global Health and Infectious Diseases Research, College of Public Health, University of South Florida, 3720 Spectrum Blvd, Tampa, Florida 33612, USA.

*Address Correspondence to Jun Miao, jmiao1@usf.edu

[§]Present address: Mohammad Kalamuddin, Parasite Cell Biology, International Centre for Genetic Engineering and Biotechnology, New Delhi, India

[#]Mohammad Kalamuddin, Ahmad Rushdi Shakri, and Chengqi Wang contributed equally to this work. Author order was determined alphabetically.

**Figure S1. Tagging of PfEAF2 with GFP tag. A**. A schematic diagram shows PfEAF2 C-terminal tagging with GFP by a single cross-over recombination strategy. hDHRF, human DHFR drug selection cassette; F2 and R2 indicate primer 1 and 2 binding upstream of the homologous arm and in GFP for the diagnostic PCR. **B**. Diagnostic PCR with primers F2 × R2 confirming successful GFP tagging in 2 clones (C1 and C2) with WT 3D7 as a negative control. **C**. PfDNMT2-GFP expression in the ring (R), trophozoite (T), and schizont (S) stages was detected by live imaging. Scale bar = 5 µm. Parasite nuclei were stained by Hoechst.

**Figure S2. Anti-hATM monoclonal antibodies did not recognize ATM in *P. falciparum*.** Western blot with mouse monoclonal antibody 2C1(1A1) detected multiple bands on the blot after transferring separated proteins of mixed stage asexual parasites via sodium dodecyl-sulfate polyacrylamide gel electrophoresis (SDS-PAGE). The protein marker (M) is shown on the left side and aldolase was used as loading control. There are no significant differences between 3D7 wildtype parasites and PfMYST-overexpression parasites. The arrow on the right side of blot indicates the putative position of the PfPI3K protein in the blot (the predicted molecular size of PfPI3K is 256 Kda).

**Figure S3. The transcription of *var* family genes at the early ring stage.** The transcriptional value of each *var* gene in PfMYST-OE parasites and wild type 3D7 at the early ring stage (8 h post-invasion) was shown as a bar graph based on the microarray data. Almost all *var* transcriptions, especially the transcriptions at high levels, in PfMYST-OE parasites were higher than wild-type parasites.

**Figure S4. Knockdown of *PfMYST* by CRISPRi.** *PfMYST* transcription was measured by RT-qPCR from the CRISPRi parasite line expressing gRNA1 targeting *PfMYST* 5' UTR (see details in Liang X. et al Microbio Spectr. 2022, 10(3):e0278221. doi:10.1128/spectrum.02782-21). The knockdown was induced by adding rapamycin to the ring-stage parasites and RNA was harvested 40h later for RT-qPCR. Three replicates (Rep) of RT-qPCR were conducted, showing ~30% reduction of *PfMYST* expression compared to the DMSO-treated parasites.

**Table S1**. List of identified proteins from TAP of PfMYST-associated proteins.
**Table S2**. List of identified proteins from GFP-trap of PfEAF2 associated proteins.
**Table S3**. Expression microarray of PfMYST-OE compared to WT during IDC.
**Table S4**. Primers used in this study.

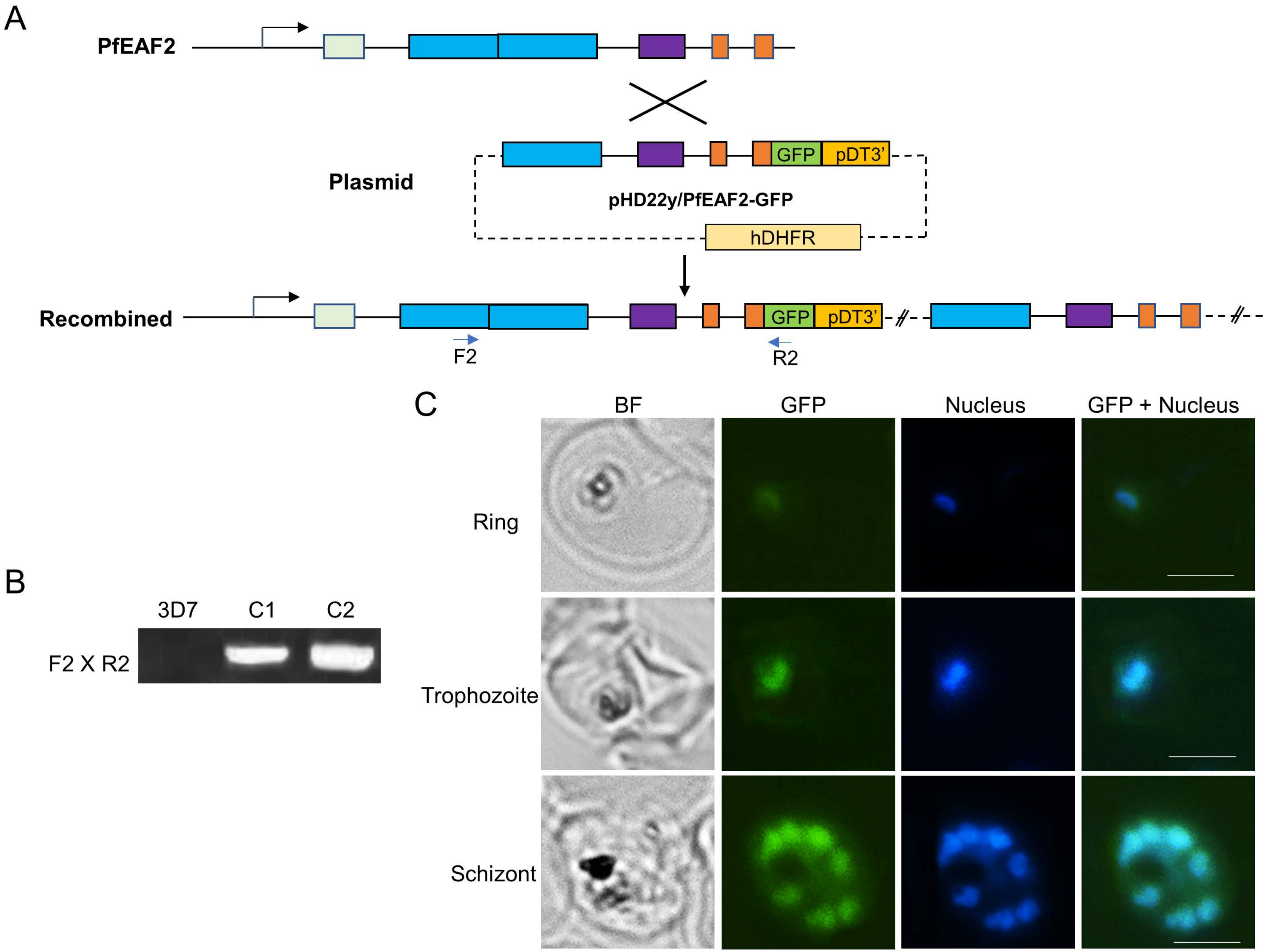


**Figure S1. Tagging of PfEAF2 with GFP tag. A**. A schematic diagram shows PfEAF2 C-terminal tagging with GFP by a single cross-over recombination strategy. hDHRF, human DHFR drug selection cassette; F2 and R2 indicate primer 1 and 2 binding upstream of the homologous arm and in GFP for the diagnostic PCR. **B**. Diagnostic PCR with primers F2 × R2 confirming successful GFP tagging in 2 clones (C1 and C2) with WT 3D7 as a negative control. **C**. PfEAF2-GFP expression in the ring (R), trophozoite (T), and schizont (S) stages was detected by live imaging. Scale bar = 5 μm. Parasite nuclei were stained by Hoechst.

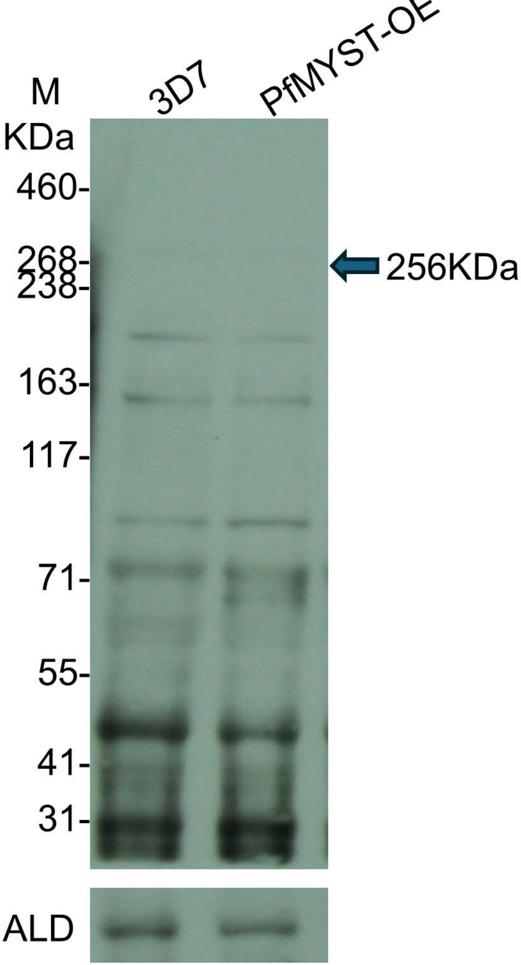


**Figure S2. Anti-hATM monoclonal antibodies did not recognize ATM in *P. falciparum*.** Western blot with mouse monoclonal antibody 2C1(1A1) detected multiple bands on the blot after transferring separated proteins of mixed stage asexual parasites via sodium dodecyl-sulfate polyacrylamide gel electrophoresis (SDS-PAGE). The protein marker (M) is shown on the left side and aldolase was used as loading control. There are no significant differences between 3D7 wildtype parasites and PfMYST-overexpression parasites. The arrow on the right side of blot indicates the putative position of the PfPI3K protein in the blot (the predicted molecular size of PfPI3K is 256 Kda).

Figure S3

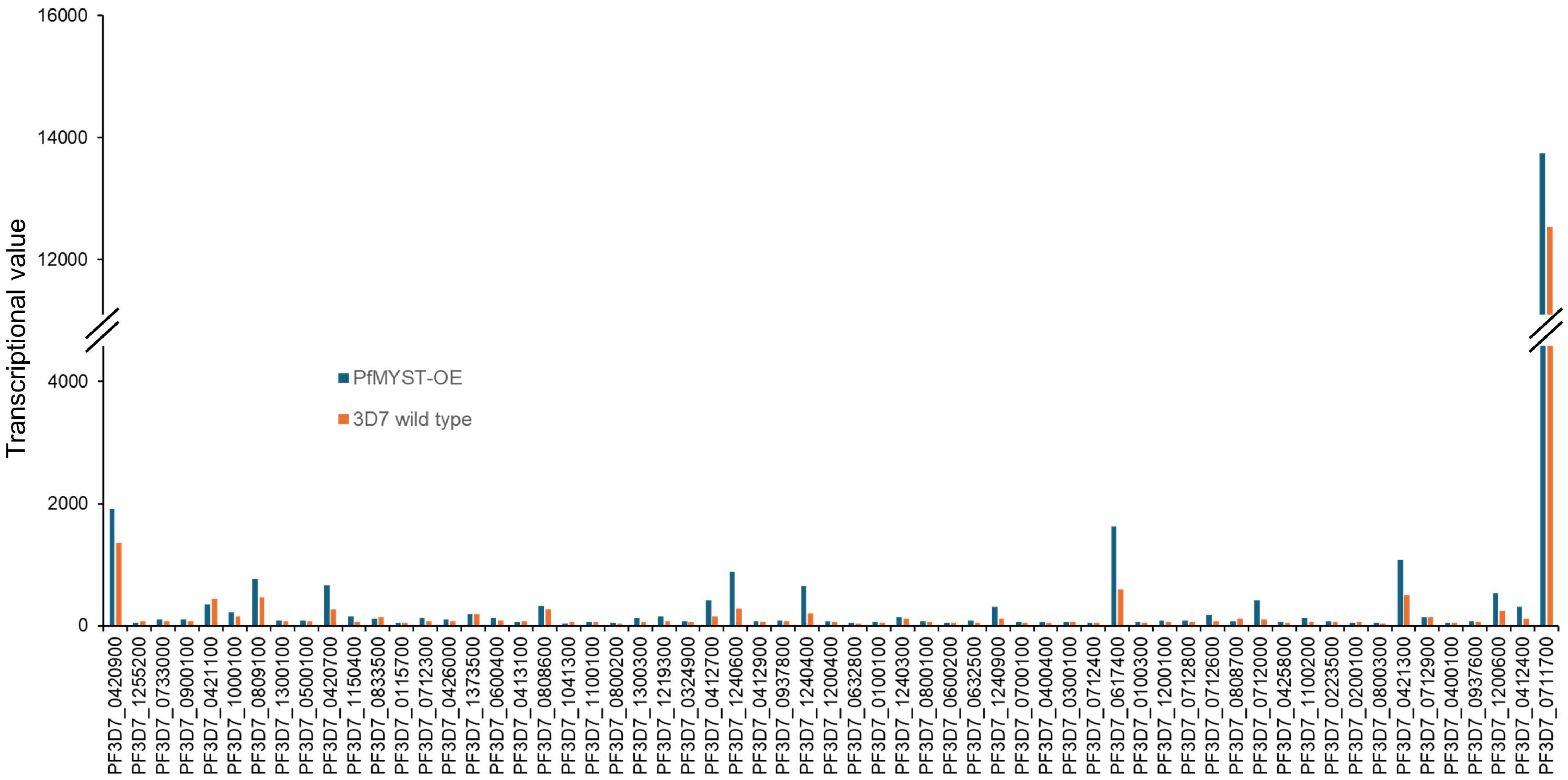


**Figure S3. The transcription of *var* family genes at the early ring stage.** The transcriptional value of each *var* gene in PfMYST-OE parasites and wild type 3D7 at early ring stage (8 h post-invasion) were showed as bar graph based on the microarray data. Almost all *var* transcriptions, especially the transcriptions at high levels, in PfMYST-OE parasites were higher than wild type parasites.

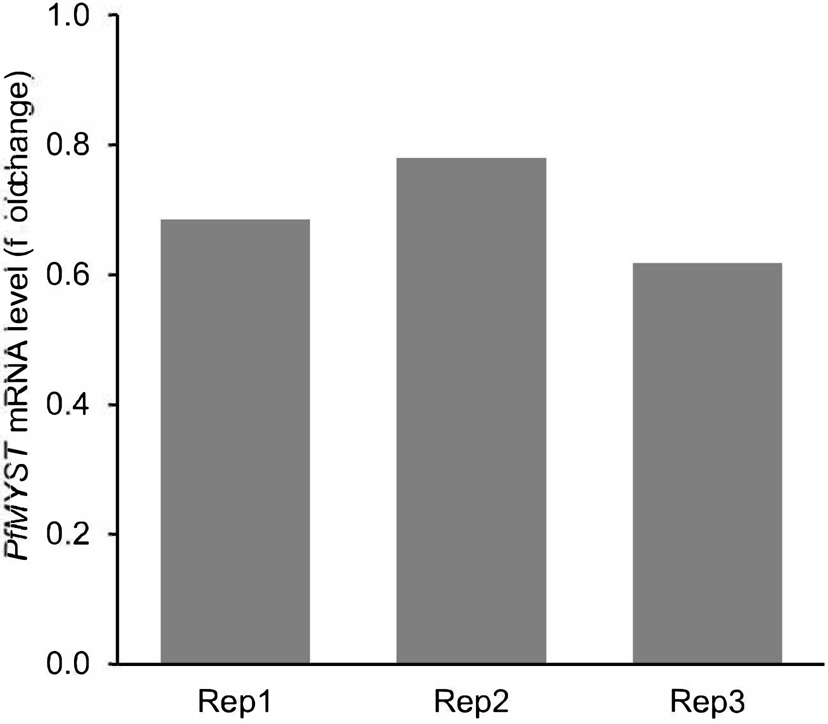


**Figure S4. Knockdown of PfMYST by CRISPRi.**
PfMYST transcription was measured by RT-qPCR from the CRISPRi parasite line expressing gRNA1 targeting PfMYST 5' UTR (see details in Liang X. et al Microbio Spectr. 2022, 10(3):e0278221. doi:10.1128/spectrum.02782-21). The knockdown was induced by adding rapamycin to the ring-stage parasites and RNA was harvested 40h later for RT-qPCR. Three replicates (Rep) of RT-qPCR were conducted, showing ~30% reduction of PfMYST expression compared to the DMSO-treated parasites.
